# Static Prognostic Factors and Appropriate Surgical Designs for Patients with Medullary Thyroid Carcinoma: The Second Report from a Single-Institution Study in Japan

**DOI:** 10.1007/s00268-018-4738-z

**Published:** 2018-07-26

**Authors:** Yasuhiro Ito, Akira Miyauchi, Minoru Kihara, Takuya Higashiiyama, Mitsuhiro Fukushima, Akihiro Miya

**Affiliations:** Department of Surgery, Kuma Hospital, 8-2-35, Shimoyamate-dori, Chuo-ku, Kobe, Hyogo 650-0011 Japan

## Abstract

**Background:**

Medullary thyroid carcinoma (MTC) originates from calcitonin-producing cells of the thyroid. In 2009, we published our first report on the biological characteristics and prognosis of 118 MTC patients. Herein, we enrolled a larger number of patients with longer follow-up periods to further study the biological characteristics and appropriate therapies for MTC.

**Methods:**

In general, hemithyroidectomy and total thyroidectomy were performed for sporadic MTC confined to the thyroid lobe and for hereditary MTC with central node dissection, respectively. Moreover, prophylactic modified radical neck dissection was performed on the side of macroscopic tumors.

**Results:**

In total, 233 patients (99 hereditary and 134 sporadic) were enrolled. The median follow-up time was 128 months (range 7–445 months). Biochemical cure was obtained in 36 (62%) of the 58 patients who underwent prophylactic MND and were pathologically positive for lateral node metastasis. None of the patients had recurrence in the preserved thyroid. Distant recurrence was detected in 19 patients, and 12 died of MTC. Preoperative calcitonin and carcinoembryonic antigen levels, tumor size (*T*) > 4 cm, the male sex, clinical and pathological node metastases (N1), distant metastasis (M1), extrathyroid extension (Ex), and a lack of biochemical cure had prognostic impacts on distant recurrence and/or carcinoma-related mortality on univariate analysis. On multivariate analysis, Ex was independently correlated with distant recurrence, and Ex, *T* > 4 cm, and M1 independently affected carcinoma-related mortality.

**Conclusion:**

MTC patients had excellent prognosis in our institutions, indicating that our surgical strategies were appropriate.

## Introduction

Medullary thyroid carcinoma (MTC) originates from calcitonin-producing cells (C-cells) of the thyroid. This carcinoma is rare and accounts for only 1.4 and 3–10% of all thyroid malignancies in Japan and in Western countries, respectively [1–5]. The typical characteristics of MTC include elevated serum calcitonin and carcinoembryonic antigen (CEA) levels, which contribute to diagnosis. Moreover, approximately 25% patients had hereditary MTC based on the germline *RET* gene mutations [6].

Hereditary MTC consists of three types, namely multiple endocrine neoplasia type 2A (MEN 2A), MEN 2B, and familial MTC (FMTC). MEN 2A is often associated with pheochromocytoma and hyperparathyroidism, while MEN 2B is associated with pheochromocytoma, ganglioneuromas, and marfanoid habitus [7–10]. On the other hand, in patients with FMTC, no coexisting endocrinopathies or neural abnormalities have been detected. Previous studies have shown that sporadic MTC has a poorer prognosis than hereditary MTC, although MEN 2B has a dire prognosis [11–14].

To date, several static prognostic factors have been identified, including the male sex, a large tumor size, extrathyroid extension (Ex), and the lack of a biochemical cure [3, 11–24]. Conversely, Miyauchi et al. proposed postoperative calcitonin-doubling time as a dynamic prognostic factor. They demonstrated that among all prognostic factors, calcitonin-doubling time had the highest correlation with 3-year survival and 5-year recurrence, making it a useful indicator for the quantitative prediction of the prognosis of MTC patients [25].

In Japan, one nationwide study about the prevalence and prognosis of MTC patients was published in [26]. In 2009, we published a single-institution study showing that lymph node metastasis, large tumor size, Ex, and the lack of a biochemical cure significantly affected carcinoma recurrence and carcinoma-related mortality [27]. Thereafter, studies enrolling a large number of MTC patients were also published from Korea [28–31].

This is the second study to date regarding MTC treated in our institution. We enrolled a larger number of patients and conducted a long-term postoperative follow-up to further investigate the clinicopathological characteristics of and appropriate surgical strategy for MTC.

## Materials and methods

### Patients

Between 1975 and 2014, 233 patients diagnosed with MTC on postoperative pathological examination and subsequently treated were enrolled in this study. No pediatric cases were included, nor were patients who underwent prophylactic total thyroidectomy. Initial systematic surgery was performed in Kuma Hospital for 224 patients, Kagawa Medical University for seven patients, and Osaka University Medical School for two patients in whom a second surgery for recurrence was performed in Kuma Hospital. All patients, with the exception of one, were preoperatively diagnosed as having MTC based on cytology and elevated calcitonin and/or CEA levels. *RET* gene mutation analysis was performed for all patients, with the analysis performed preoperatively and postoperatively for patients treated before and after November 1995, respectively. We measured the metanephrine and normetanephrine in the urine and conducted imaging examinations, for example computed tomography (CT) scans in order to detect the pheochromocytoma for RET gene mutation-positive cases. Surgery for pheochromocytoma was performed prior to the treatment of MTC if pheochromocytoma was detected using these tests.

Twelve patients with *RET* gene mutations underwent prophylactic total thyroidectomy, although carcinoma in the thyroid and lymph nodes was not detected on preoperative imaging evaluations. The presence of MTC was confirmed for these patients using pathological examinations. Patients with only C-cell hyperplasia were excluded from this study. All study participants provided informed consent, and since this was a retrospective study, approval by our ethics review board was not required.

### Surgical designs

Before 1996, we usually performed total thyroidectomy in all MTC cases. However, after *RET* gene mutation analysis became available, we switched to hemithyroidectomy [lobectomy with isthmectomy or subtotal thyroidectomy (dissecting one lobe, isthmus, and the lower pole of the contralateral lobe)] for MTC patients without *RET* gene mutations [27, 32, 33] whose tumors are solitary and located in one lobe. Lobectomy with isthmectomy was also performed when pathological lesions were located in the isthmus. If tumors were large and occupied one lobe, isthmus, and part of the contralateral lobe, we performed a total thyroidectomy. We evaluated whether the clinical node metastasis was positive by conducting imaging evaluations, including ultrasounds and CT scans. All patients underwent routine central node dissection (CND), with the exception of four patients with hereditary MTC without apparent carcinoma lesions based on ultrasound findings. Modified radical neck dissection (MND) at the side of macroscopic tumors was also routinely performed although it was prophylactic. MND was also performed for patients with clinical nodal metastasis in the lateral compartment. Upper mediastinal dissection was performed as necessary.

### Postoperative follow-up

Approximately 1 week after surgery, we measured calcitonin levels before and after stimulation with calcium gluconate, with or without tetragastrin to investigate whether patients were biochemically cured; the procedure for measurement was as described previously [25]. In the era of patients enrolled in this study, calcitonin was measured using the solid two-site immunoradiometric assay, although the assay was changed to the electrochemiluminescence immunoassay since April 2015 [34, 35].

CEA was measured via the radioimmune assay (RIA) until September 2000 (normal value ≤2.5 ng/ml). Thereafter, the assay technique was changed to the chemiluminescent immunoassay (CLIA) until December 2006 and then the chemiluminescent enzyme immunoassay (CLEIA) from December 2006 onwards, which is currently in use (normal value ≤5.0 ng/ml). In this study, we multiplied the CEA values obtained via RIA by two to match the data to those obtained via CLIA and CLEIA for statistical analyses. Serum CEA and calcitonin were measured 2–4 times per year after surgery. In patients with elevated serum CEA and calcitonin levels, particularly in those whose calcitonin-doubling time became shorter, imaging evaluations, such as ultrasonography, CTs, and positron emission-CTs, were performed to detect recurrent lesions. Fine-needle aspiration cytology (FNAC) is the standard diagnostic modality for local recurrence. In particular, suspicious lesions were evaluated by measuring calcitonin on the washout of the needles used for FNAC in recent cases [36].

Re-operation was usually the preferred treatment for local recurrence, including recurrence to the upper mediastinal lymph nodes. Meanwhile, extrabeam radiotherapy was performed for distant recurrence and inoperable recurrent tumors in the neck according to the attending physician’s discretion. Two patients (one M1 patient and another patient who showed distant recurrence after surgery) underwent tyrosine kinase inhibitor (TKI) therapies (vandetanib and sorafenib, respectively).

For patients who stopped visiting our hospital, a questionnaire was sent once yearly to assess their condition.

### Statistical analyses

Fisher’s exact tests were used to compare categorical variables. The Kaplan–Meier and log-rank tests were used for the analysis of time-dependent variables. Multivariate analysis was performed using a Cox regression model and log-rank test. All statistical analyses were performed using StatView, and *p* values < 0.05 and 0.05–0.1 were considered to indicate significance and strong tendencies, respectively.

## Results

Based on the germline *RET* gene mutation analysis, 99 of the 233 patients had hereditary MTC, while the remaining 134 had sporadic MTC. The median follow-up time of the enrolled participants was 128 months (range 7–445 months). Table 1 shows the comparison of the demographic and clinicopathological features of the patients with hereditary and sporadic MTC. Total thyroidectomy was performed in 46 (34%) and 96 (97%) patients with sporadic and hereditary MTC, respectively. In our series, CND and prophylactic MND at the side of macroscopic tumors were typically performed, except for patients with tumors smaller than 1 cm, who normally underwent CND only. We performed therapeutic MND for 48 patients, while prophylactic MND was performed for 164 patients. Of 48 patients who underwent therapeutic MND, two (4%) showed chyle leakage with a conservative cure, one (2%) showed chyle leakage requiring re-operation, and one (2%) suffered vagus nerve injury. Of 164 who underwent prophylactic MND, in contrast, only two (1.2%) showed chyle leakage which was cured by the consumption of a fat-restricted diet.

**Table 1:** Relationships between *RET gene* mutations and various clinicopathological features

	*RET* mutations		Total (*N *= 233) (100%)	*p* value
	Positive	Negative		
	(*N* = 99) (100%)	(*N *= 134) (100%)		
Age (years)	39.9 ± 18.5	54.9 ± 13.9		<0.001
Sex				0.653
Male	27 (27%)	33 (25%)	60 (26%)	
Female	72 (73%)	101 (70%)	173 (74%)	
Thyroidectomy				
Lobectomy with isthmectomy	0	65 (49%)	65 (28%)	<0.001
Subtotal	3 (3%)	23 (17%)	26 (11%)	
Total	96 (97%)	46 (34%)	142 (61%)	
Lymph node dissection				
Not done	3 (3%)	1 (1%)	4 (2%)	<0.001
Central only	16 (16%)	1 (1%)	17 (7%)	
Central + MND^a^	80 (81%)	132 (98%)	212 (91%)	
Tumor size				
>4 cm	6 (6%)	15 (11%)	21 (9%)	0.191
≤4 cm	91 (94%) (unknown 2)	119 (89%)	210 (91%)	
cN				
cN1	21 (21%)	29 (22%)	50 (21%)	0.937
cN0	78 (79%)	105 (78%)	183 (79%)	
pN				
pN1	51 (52%)	71 (53%)	122 (53%)	0.887
pN0	47 (48%) (unknown 1)	63 (47%)	110 (47%)	
Extrathyroid extension^b^				
Yes	8 (8%)	7 (5%)	15 (6%)	0.380
No	91 (91%)	127 (95%)	218 (94%)	
Biochemical cure				
Yes	69 (69%)	91 (68%)	160 (69%)	0.771
No	30 (30%)	43 (32%)	73 (31%)	
Multiplicity based on histopathological findings				
Yes	86 (88%)	24 (18%)	110 (47%)	<0.001
No	12 (12%)^c^ (unknown 1)	110 (82%)	122 (53%)	
Multiplicity to both lobes based on histopathological findings^d^				
Yes	59 (62%)	1 (2%)	60 (43%)	
No	36 (38%)	45 (98%)	81 (57%)	< 0.001
M				
M1	2 (2%)	2 (1%)	4 (2%)	
M0	97 (97%)	132 (99%)	229 (98%)	0.759
Stage (8th UICC/AJCC staging system)^e^				
I	41 (48%)	57 (44%)	98 (45%)	0.792
II	25 (29%)	42 (31%)	67 (30%)	
III	2 (2%)	7 (5%)	9 (4%)	
IVA	16 (19%)	26 (19%)	42 (19%)	
IVC	2 (2%)	2 (1%)	4 (2%)	

*MND* modified radical neck dissection

^a^Uni- or bilateral MND; eight patients also underwent upper mediastinal dissection

^b^Minimal or significant extension based on intraoperative findings

^c^Of these, C-cell hyperplasia was detected on histopathological examination in seven patients

^d^Patients who underwent total thyroidectomy were enrolled

^e^Based on the 8th UICC/AJCC TNM staging system; 13 patients who underwent prophylactic total thyroidectomy were excluded

Herein, 86 of the 98 (88%) patients with hereditary MTC (one unknown) had multiple tumors on pathological findings. Meanwhile, of the 12 patients pathologically diagnosed with solitary tumors, seven had C-cell hyperplasia (Table 1). The incidence of multiplicity was significantly lower (*p* < 0.001) at 18% in the sporadic MTC group. Of the 46 patients with sporadic MTC who underwent total thyroidectomy, only one (2%) had tumor multiplicity on both thyroid lobes. This patient was diagnosed with multiple MTC on preoperative ultrasound that was exceptionally aggressive and showed clinically extensive lymph node metastasis at central and bilateral lateral components. Although curative surgery was performed, this patient died of MTC only 7 months after surgery.

Table 2 shows the relationship between the postoperative biochemical cure and the clinicopathological features of patients. High preoperative calcitonin levels (≥1000 pg/ml), high CEA levels (≥20 ng/ml), large tumor sizes (>4 cm), cN1, Ex, and M1 were inversely related to the biochemical cure. Of the 163 patients who tested negative for clinical lateral node metastasis but underwent prophylactic MND, 58 (36%) were lateral node positive on pathological examination. Of these 58 patients, 36 (62%) achieved biochemical cure. The incidence of carcinoma death in patients who were not biochemically cured was significantly higher than that in patients who were biochemically cured (Table 2). However, of the 73 patients who were not biochemically cured, including four M1 patients and one who underwent local non-curative surgery, only 10 (14%) died with MTC during the study period.

**Table 2:** Relationships between biochemical cure and various clinicopathological features

	Biochemical cure		Total	*p* value
	Yes (*N* = 160)	No (*N* = 73)		
Sex				0.197
Male	37 (62%)	23 (38%)	60 (100%)	
Female	123 (71%)	37 (29%)	173 (100%)	
Preoperative CEA level				<0.001
High (≥20 pg/ml)	65 (53%)	57 (47%)	122 (100%)	
Low (<20 pg/ml)	94 (89%) (unknown 1)	12 (11%) (unknown 4)	106 (100%) (unknown 5)	
Preoperative calcitonin level				<0.001
High (≥1000 ng/ml)	74 (55%)	60 (45%)	134 (100%)	
Low (<1000 ng/ml)	86 (87%)	13 (13%)	99 (100%)	
*RET* gene mutations				0.887
Yes	69 (70%)	30 (30%)	99 (100%)	
No	91 (68%)	43 (32%)	134 (100%)	
Tumor size				
>4 cm	11 (52%)	10 (48%)	21 (100%)	0.087
≤4 cm	149 (71%)	61 (29%) (unknown 2)	210 (100%)	
cN				
cN1	10 (25%)	40 (75%)	50 (100%)	<0.001
cN0	150 (82%)	33 (18%)	183 (100%)	
pN				
pN1	53 (43%)	69 (57%)	122 (100%)	0.887
pN0	106 (96%) (unknown 1)	4 (4%)	110 (100%)	
Extrathyroid extension^a^				
Yes	4 (27%)	11 (73%)	15 (100%)	0.001
No	156 (72%)	62 (28%)	218 (100%)	
M				
M1	0 (0%)	4 (100%)	4 (100%)	
M0	160 (70%)	69 (30%)	229 (100%)	<0.001
Stage (8th UICC/AJCC staging system)^b^				
I	80 (82%)	18 (18%)	98 (100%)	<0.001
II	51 (76%)	16 (24%)	67 (100%)	
III	7 (78%)	2 (22%)	9 (100%)	
IVA	10 (24%)	32 (76%)	42 (100%)	
IVC	0 (0%)	4 (100%)	4	
Carcinoma death				
Yes	2 (17%)	10 (83%)	12 (100%)	<0.001
No	158 (71%)	63 (26%)	221 (100%)	

*CEA* carcinoembryonic antigen

^a^Minimal or significant extension based on intraoperative findings

^b^Based on the 8th UICC/AJCC TNM staging system; 13 patients who underwent prophylactic total thyroidectomy were excluded

Table 3 shows the relationship between pathological lateral node metastasis and various clinicopathological features for 164 patients with MTC who underwent prophylactic MND. Patients with tumor multiplicity, based on preoperative imaging evaluations, had more frequent lateral node positivity based on pathological examination than those without tumor multiplicity (*p* = 0.018). Preoperative CEA and calcitonin levels were significantly (*p* = 0.0209) and marginally (*p* = 0.0968) related to pathological lateral node metastasis. However, 28% of solitary MTC based on the preoperative findings were pathologically positive for lateral node metastasis, while 25 and 26% of MTC with low CEA and calcitonin levels, respectively, had metastasis in the lateral node. Twenty-nine percent of the MTC measuring 2 cm or less were lateral node positive. Therefore, the incidence of lateral node metastasis in MTC patients was still high even though the MTC showed no aggressive features.

**Table 3:** Relationships between pathological lateral node metastasis and clinicopathological features in 164 patients with medullary thyroid carcinoma who underwent prophylactic lateral node dissection

	Pathological N1b		Total	*p* value
	Yes (*N* = 55)	No (*N* = 109)		
Sex				0.570
Male	16 (38%)	26 (62%)	42 (100%)	
Female	39 (32%)	83 (68%)	122 (100%)	
Preoperative CEA level				0.0209
High (≥20 pg/ml)	34 (43%)	46 (57%)	80 (100%)	
Low (<20 pg/ml)	21 (25%)	63 (75%)	84 (100%)	
Preoperative calcitonin level				0.0968
High (≥1000 ng/ml)	35 (40%)	53 (60%)	88 (100%)	
Low (<1000 ng/ml)	20 (26%)	56 (74%)	76 (100%)	
*RET* gene mutations				0.607
Yes	22 (37%)	38 (63%)	60 (100%)	
No	33 (31%)	71 (69%)	104 (100%)	
Tumor size (cm)				
>4	5 (45%)	6 (55%)	11 (100%)	0.099
3.1–4	5 (21%)	19 (79%)	24 (100%)	
2.1–3	18 (45%)	22 (55%)	40 (100%)	
≤2	26 (29%)	63 (71%)	89 (100%)	
Multiplicity based on preoperative findings				
Yes	23 (48%)	25 (52%)	48 (100%)	0.018
No	32 (28%)	84 (72%)	116 (100%)	

*CEA* carcinoembryonic antigen

Table 4 indicates the organs in which MTC recurred. To date, 29 patients showed MTC recurrence in various organs, and of these, 19 showed recurrence in distant organs (13 patients had both local and distant recurrence). Importantly, none of the patients who underwent hemithyroidectomy developed recurrence in the preserved thyroid lobe.

**Table 4:** Organs in which medullary thyroid carcinoma recurred

*N* = 29 (100%)	
Local organs	
Lymph nodes	23
Preserved thyroid	0
Distant organs	
Lung	10
Liver	5
Bone	9

Twelve patients showed recurrences in two or more organs. The organ in which one patient developed recurrence is unknown

We excluded 12 patients who underwent prophylactic total thyroidectomy and were diagnosed with hereditary MTC (T0 cases) based on the analyses of prognosis and prognostic factors, because this condition has an excellent outcome. Of the remaining 221 patients, four M1 patients and one patient who underwent local non-curative surgery (some upper mediastinal lymph nodes remained unresected) were excluded from the analysis of distant recurrence-free survival (DRFS), but included in the cause-specific survival (CSS) analysis.

**Fig. 1:**
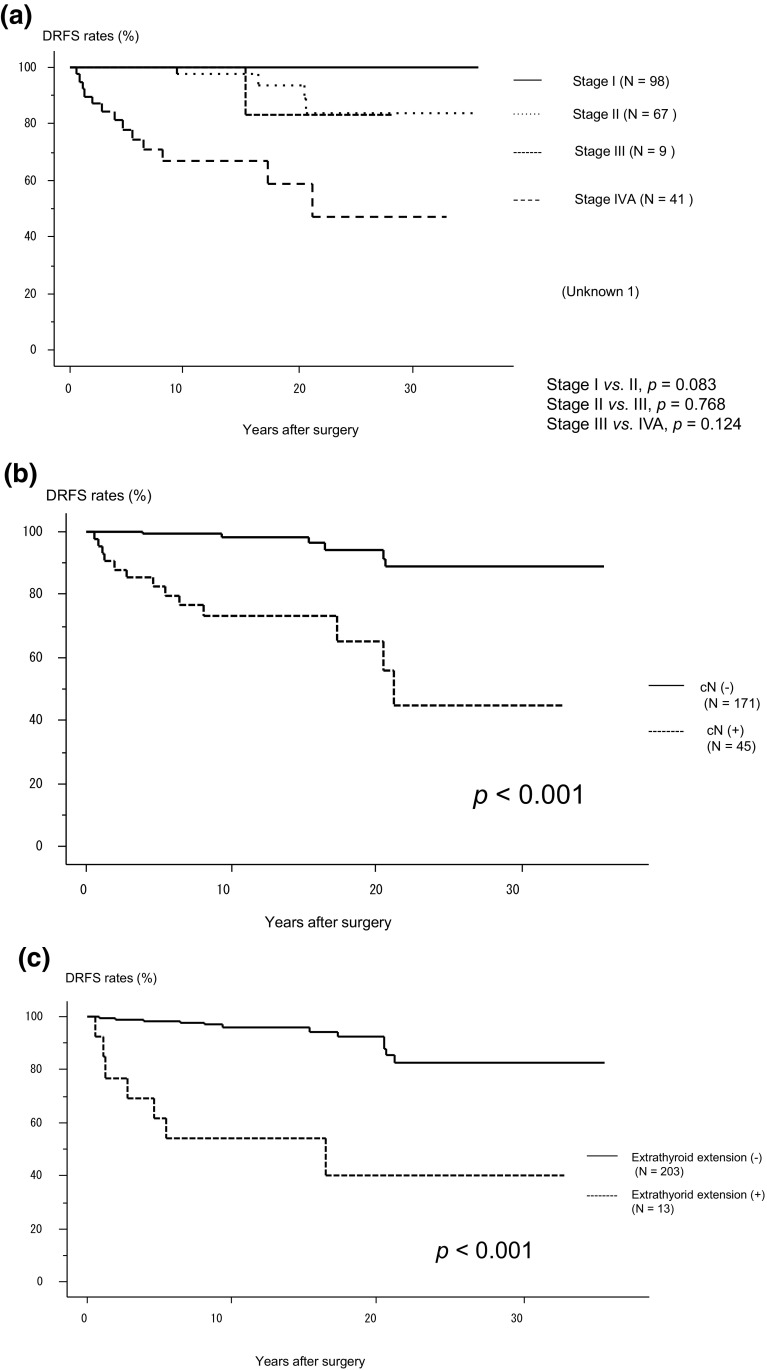
**a** Kaplan–Meier curves for distant recurrence-free survival of medullary thyroid carcinoma patients belonging to each stage (I–IVA). **b** Kaplan–Meier curves for distant recurrence-free survival of patients with and without clinical node metastasis. **c** Kaplan–Meier curves for distant recurrence-free survival of patients with and without extrathyroid extension

The 5-, 10- and 20-year DRFS rates were 96.1, 93.5 and 88.5%, respectively. Figure 1a shows the DRFS of MTC patients in Stages I–IVA as classified according to the 8th edition of the AJCC/UICC TNM staging system.

The DRFS of Stage II patients tended to be worse (*p *= 0.083) than that of Stage I patients, but the DRFS of Stage II and III patients was similar. Meanwhile, the DRFS of Stage IVA patients was worse than that of Stage III patients, but the difference was not significant (*p *= 0.124) (Fig. 1a). We then investigated the clinicopathological factors for predicting distant recurrence. Patients with high preoperative calcitonin (*p *= 0.078) and CEA levels (*p *= 0.047), with tumors larger than 4 cm (*p *= 0.051), and who were cN (*p *< 0.001) (Fig. 1b) and pN positive (*p *= 0.003), did not achieve biochemical cure (*p *< 0.001). Those who were Ex positive (*p *< 0.001) (Fig. 1c) frequently showed distant recurrence, while *RET* mutations (*p *= 0.894) and sex (*p *= 0.205) did not affect the rate of distant recurrence.

Table 5 shows the multivariate analysis of the various factors for DRFS. Although Ex was an independent prognostic factor of DRFS (*p* < 0.001, OR 12.82), its incidence rate was low at 6%. We then excluded Ex and performed the multivariate analysis again. Thereafter, we found that cN persisted as an independent predictor of DRFS (*p *= 0.016, OR 4.878).

**Table 5:** Multivariate analysis of factors affecting distant recurrence-free survival in medullary thyroid carcinoma patients

Variable	*p* value	OR (95% CI)
Male sex	0.114	2.667 (0.862–8.196)
Preoperative calcitonin ≥1000 pg/ml	0.164	5.291 (0.507–55.556)
Preoperative CEA ≥20 ng/ml	0.178	3.406 (0.571–20.320)
*RET* mutations (–)	0.648	0.775 (0.261–2.304)
Tumor size >4 cm	0.426	1.739 (0.445–6.802)
cN (+)	0.268	2.174 (0.551–8.547)
Extrathyroid extension (+)	<0.001	12.820 (3.333–47.619)
Biochemical cure (–)	0.114	3.028 (0.802–11.428)

*CI* confidence interval, *CEA* carcinoembryonic antigen

**Fig. 2:**
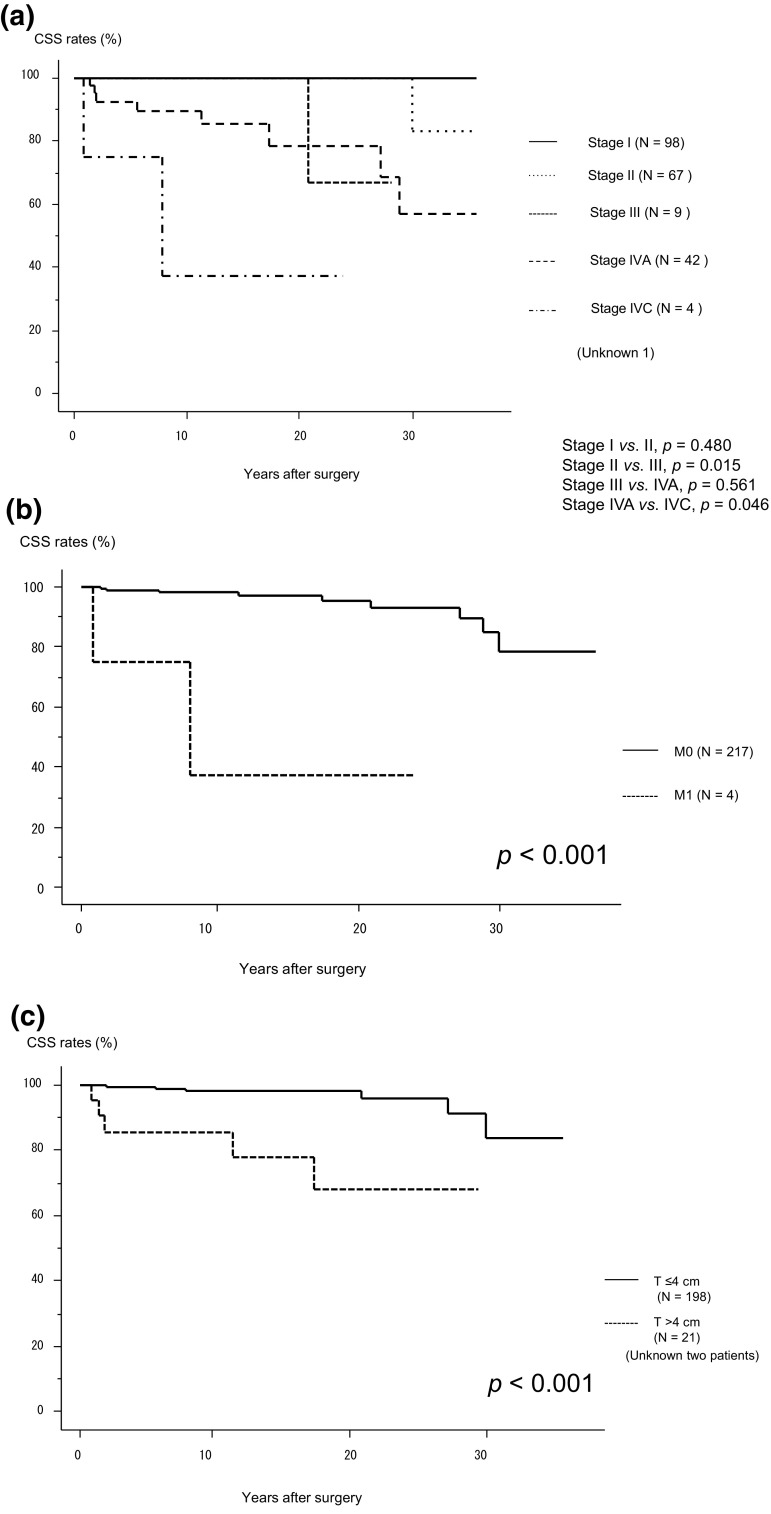

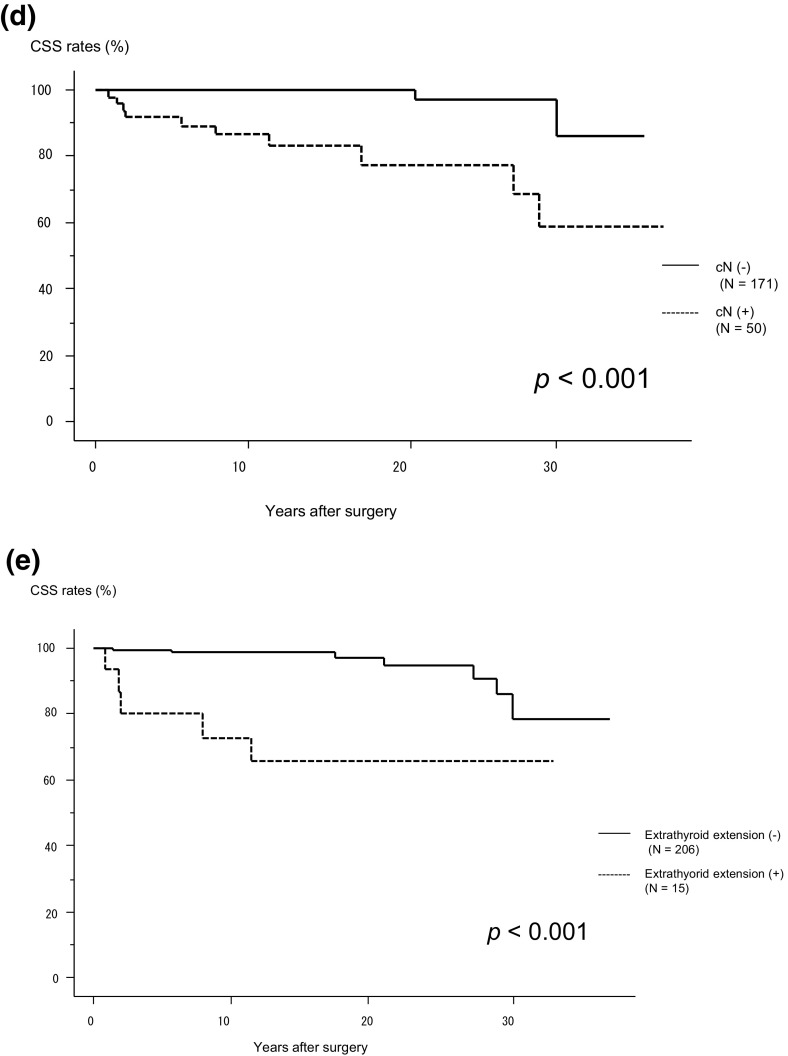
**a** Kaplan–Meier curves for cause-specific survival of medullary thyroid carcinoma patients belonging to each stage (I–IVA and IVC). **b** Kaplan–Meier curves for cause-specific survival of M1 and M0 patients. **c** Kaplan–Meier curves for cause-specific survival of patients with tumors measuring ≤4 cm and >4 cm. **d** Kaplan–Meier curves for cause-specific survival of patients with and without clinical node metastasis. **e** Kaplan–Meier curves for cause-specific survival of patients with and without extrathyroid extension. *CSS* cause-specific survival

We then analyzed the CSS of 221 patients. Over the study period, 12 patients died of MTC, and the 5-, 10-, and 20-year CSS rates were 98.2, 97.0, and 94.3%, respectively. As shown in Fig. 2a, the CSS of Stage II patients did not differ from that of Stage I patients, while Stage III patients had a significantly worse CSS than Stage II patients (*p *= 0.015). The CSS of Stage IVA patients did not differ from that of Stage III patients, and Stage IVC patients had a significantly worse CSS (*p *= 0.046) than Stage IVA patients. On univariate analysis, CSS was significantly related to M1 positivity (*p *< 0.001) (Fig. 2b), tumors larger than 4 cm (*p *< 0.001) (Fig. 2c), the male sex (*p *= 0.022), cN positivity (*p *< 0.001) (Fig. 2d), pN positivity (*p *= 0.020), the presence of Ex (*p *< 0.001) (Fig. 2e), and the lack of a biochemical cure (*p *= 0.007). However, the preoperative calcitonin (*p *= 0.402) and CEA levels (*p *= 0.255) and *RET* gene mutations (*p *= 0.474) did not affect CSS.

On multivariate analysis, tumors larger than 4 cm (*p *= 0.039, OR 5.650) and Ex (*p *= 0.034, OR 8.850) were independent predictors of carcinoma death (Table 6). Although the *p* value was not <0.05, M1 was regarded to highly likely affect the CSS of patients (*p *= 0.051, OR 9.174). Only 2 and 6% patients were M1 and Ex positive, respectively. We then excluded these two factors from the analysis. Thereafter, we found that tumors larger than 4 cm (*p *= 0.007, OR 7.042) and clinical node metastasis (*p *= 0.024, OR 14.49) independently predicted the CSS of patients.

**Table 6:** Multivariate analysis of factors affecting CSS in medullary thyroid carcinoma patients

Variable	*p* value	OR (95% CI)
Male sex	0.476	1.841 (0.085–2.062)
Preoperative calcitonin ≥1000 pg/ml	0.775	0.693 (0.057–8.475)
Preoperative CEA ≥20 ng/ml	0.212	0.144 (0.007–3.012)
*RET* mutations (–)	0.285	2.383 (0.485–11.696)
Tumor size >4 cm	0.039	5.650 (1.088–29.412)
cN (+)	0.192	6.579 (0.581–71.429)
Extrathyroid extension (+)	0.034	8.850 (1.179–66.667)
Biochemical cure (–)	0.340	3.329 (0.254–43.668)
Distant metastasis (+)	0.051	9.174 (0.993–83.333)

*CSS* cause-specific survival, *MTC* medullary thyroid carcinoma, *CI* confidence interval, *CEA* carcinoembryonic antigen

Lastly, we performed a subset analysis of 88 patients with sporadic MTC who underwent hemithyroidectomy. The median follow-up period was 120 months (range 7–374 months). One patient had multiple lung metastases but was still alive 42 months after surgery. Distant recurrence was detected in four patients at 47, 98, 184, and 198 months after surgery, although 20 patients were not biochemically cured. Only two patients died of MTC at 208 and 250 months after surgery.

## Discussion

In this study, we demonstrated that the 10- and 20-year CSS rates of MTC patients were 97.0 and 94.3%, respectively, which are better than those from our first report, which were 96.6 and 91.7%, respectively [27]. The incidences of aggressive tumors were higher in the previous series than those in the present study: cN1 (23% [26/115] vs. 21% [50/233]), Ex (11% [13/115] vs. 6% [15/233]), tumors larger than 4 cm (12% [13/113 (two unknown)] vs. 9% [21/231 (two unknown)]), and pN1 (58% [67/115] vs. 53% [122/232 (one unknown)]) [27]. The decrease in aggressive cases might be attributed to the early detection of thyroid nodules, including MTC, from the enhanced diagnostic capability such as the development of ultrasounds and the improvement in the accuracy of FNAC. This should, in part, improve the prognosis of MTC patients. Similar results were also demonstrated in reports from Korea [28, 30].

In this study, we showed that although carcinoma multiplicity was pathologically detected in 24 (18%) of the 133 patients with sporadic MTC, only one of the 46 (2%) who underwent total thyroidectomy had a gross multiplicity on both lobes. She had an unusually aggressive malignancy and died of distant recurrence only 7 months after surgery. Except for such an unusually aggressive case, none of the patients with sporadic MTC showed multiplicity on both lobes. Importantly, none of the patients with sporadic MTC who underwent hemithyroidectomy showed recurrence in the preserved thyroid lobe. Furthermore, based on the subset analysis of 88 patients with sporadic MTC (median follow-up, 120 months), the 15-year CSS rate was 100%, and only two patients died of MTC thereafter. We can therefore conclude that, for patients with true sporadic MTC without germline *RET* mutations having a solitary MTC tumor confined in a lobe, total thyroidectomy was not needed, and hemithyroidectomy was adequate as a locally curative surgery [32, 33].

Regarding lymph node dissection, 90% of the patients underwent not only central node dissection, but also MND at the side of macroscopic tumors regardless if it is therapeutic or prophylactic. Indeed, biochemical cure was obtained in 62% patients who underwent prophylactic MND and were pathological lateral node positive. Unlike differentiated carcinoma, radioactive iodine therapy and suppression of the thyroid-stimulating hormone are ineffective for MTC. Surgery is the only initial strategy for local control. Therefore, our institution has actively performed lymph node dissection, including prophylactic MND, for MTC patients. The incidence of pathological lateral node metastasis was positively related to preoperative multiplicity and CEA levels, but 28 and 25% patients with solitary MTC and low CEA levels, respectively, were still lateral node positive. Moreover, 29% of MTCs measuring ≤ 2 cm were positive for lateral node metastasis. Therefore, it is difficult to determine the indication for prophylactic MND based on the clinicopathological features, due to the high incidence of lateral node metastasis of MTC, even though it had no aggressive features. Of course, it is debatable whether prophylactic MND is mandatory for N0 or N1a MTC patients, and it is also true that prophylactic MND is not the usual practice in the real world. Furthermore, to our knowledge no studies have compared the prognosis and the incidence of the biochemical cure of N0 or N1a between MTC patients who did and did not undergo prophylactic MND. In our institution, the incidence of prophylactic MND complication was very low, but prophylactic MND may induce some significant complications such as chylous pleural effusion, injury of the vagus nerve, and Horner syndrome, if performed by non-experts. We performed prophylactic MND according to the policy, to reduce lymph node recurrence; our study showed excellent outcomes, including low rates of morbidity. Furthermore, prophylactic MND contributed to the staging of each case. Based on our findings, prophylactic MND could be considered appropriate, but there is no evidence globally to suggest that this surgical procedure is the best.

As shown in this study, the prognosis of MTC patients was generally good. Recently, a few TKIs have been proven to be effective for advanced or recurrent MTC [37–39]. However, in our series, only 10 of the 63 patients who were not biochemically cured died of MTC, and the remaining patients survived asymptomatic for a long time, although some of them had recurrent lesions. Therefore, the indications of TKIs should be limited to patients whose unresectable recurrent lesions substantially and constantly grow. Active surveillance of calcitonin levels is very useful for evaluating the growth of recurrence. Miyauchi et al. [25] demonstrated that short calcitonin-doubling time adequately predicts the growth of recurrent lesions and carcinoma death in MTC patients. Thus, calcitonin-doubling time is an important predictive indicator of the prognosis of MTC patients and is also important in determining the suitability and appropriateness of TKIs for treatment. From our clinical experience, we believe that the current TKIs are suitable for unresectable MTCs with calcitonin-doubling time <1.5 years.

We demonstrated that large tumor size, clinical node metastases, biochemical cure, Ex, and distant metastasis at diagnosis had a significant prognostic value; these results are consistent with those from a previous study [27]. Based on multivariate analysis, Ex independently affected the DRFS of patients, and Ex, distant metastasis at presentation, and large tumor size (>4 cm) were independent predictors of carcinoma death of patients. After excluding factors that were uncommon in the cohort, such as Ex and distant metastasis at presentation, clinical node metastasis was regarded as a predictive factor of distant recurrence and carcinoma death of MTC patients on multivariate analysis. Patients with MTC with these risk factors should be carefully treated and followed after surgery.

In this study, Ex was defined as the presence of minimal or significant extension based on intraoperative findings. We investigated the difference in prognostic significance for DRFS and CSS between minimal and significant extension, but could not find any statistical difference between them (data not shown), although in papillary thyroid carcinoma, minimal extension has little prognostic value [40]. This may be because minimal or significant Ex is rather rare in MTC, and the number of patients with Ex enrolled in this study was small (eight for significant extension and seven minimal extension). This is a limitation of this study, and further studies enrolling a larger number of patients with Ex are needed. However, currently, we should conclude that minimal or significant Ex based on intraoperative findings is significantly related to the patient’s prognosis.

Previous studies showed that although hereditary MTC is generally indolent and has an excellent prognosis, MEN2B is aggressive and has poor prognosis [11–14]. Our cohort included only five patients with MEN2B. One of these patients showed both local and distant recurrence and died due to MTC 22 months after surgery. Meanwhile, the remaining four are still alive with no recurrence 34, 74 (this patient underwent prophylactic total thyroidectomy), 140 and 233 months after surgery, although the calcitonin level of one patient continued to be high. Two of these four patients, including one who underwent prophylactic total thyroidectomy, had rare germline tandem V804M/Y806C mutations [41]. In this study, we could not clearly show the difference in prognosis between patients with MEN2B and other hereditary diseases. Further studies enrolling larger number of MEN2B patients are necessary to elucidate this point.

In our study, MTC patients showed an excellent prognosis. Machens et al. [42] reported a 5-year mortality rate of 10.7%, which was the lowest rate reported. However, the prognosis of our patients was better, although stage-matched comparisons are needed for accurate analysis. The preoperative diagnostic rate of MTC based on calcitonin measurement and FNAC was high in our patients, and with the exception of one patient, they were all preoperatively diagnosed as or suspected of having MTC. After the introduction of *RET* gene mutation analysis, we routinely performed this analysis to determine the appropriate surgical strategy for sporadic and hereditary patients. Hemithyroidectomy was performed for patients with true sporadic MTC without *RET* gene mutations if the carcinoma lesion was solitary and limited in one lobe on preoperative imaging studies, while total thyroidectomy was performed for patients with hereditary MTC regardless of whether the carcinoma lesion was solitary or multiple. Additionally, prophylactic MND at the side of macroscopic tumors was performed for most cases except for T0 patients who underwent prophylactic surgery and those having small tumors (≤1 cm). These treatment modalities would largely contribute to the treatment of MTC patients and yield excellent prognosis.

## References

[1] Ezaki H, Ebihara S, Fukumoto Y (1992). Analysis of thyroid carcinoma based on material registered in Japan during 1977–1986 with special reference to predominance of papillary type. Cancer.

[2] Moley JE, DeBenedetti MK (1999). Patterns of nodal metastasis in palpable medullary thyroid carcinoma: recommendations for extent of node dissection. Ann Surg.

[3] Kebebew E, Itunarte PH, Siperstein AE (2000). Medullary thyroid carcinoma: clinical characteristics, treatment, prognostic factors, and a comparison of staging systems. Cancer.

[4] Gulben K, Berberoglu U, Boyabatli M (2006). Prognostic factors for sporadic medullary thyroid carcinoma. World J Surg.

[5] DeLellis RA, Rule AH, Spiler I (1978). Calcitonin and carcinoembryonic antigen as tumor markers in medullary thyroid carcinoma. Am J Clin Pathol.

[6] Raue R, Kkotzerke J, Reinwein D (1993). Prognostic factors in medullary thyroid carcinoma: evaluation of 741 patients from the German Medullary Thyroid Carcinoma Register. Clin Investig.

[7] Mulligan LM, Kwok JB, Healey CS (1993). Germ-line mutations of the RET proto-oncogene in multiple endocrine neoplasia type 2A. Nature.

[8] Donis-Keller H, Dou S, Chi D (1993). Mutations in the RET proto-oncogene are associated with MEN 2A and FMTC. Hum Mol Genet.

[9] Carlson KM, Dou S, Chi D (1994). Single missense mutation in the tyrosine kinase catalytic domain of the RET protoncogene is associated with multiple endocrine neoplasia type 2B. Proc Natl Acad Sci USA.

[10] Hyer SL, Newbold K, Harmer C (2005). Familial medullary thyroid cancer: clinical aspects and prognosis. Eur J Surg Oncol.

[11] Cupisti K, Wolf A, Raffel A (2007). Long-term clinical and biochemical follow-up in medullary thyroid carcinoma: a single institution’s experience over 20 years. Ann Surg.

[12] Lee NC, Norton JA (2000). Multiple endocrine neoplasia type 2B—genetic basis and clinical expression. Surg Oncol.

[13] Brauckhoff M, Gimm O, Weiss CL (2004). Multiple endocrine neoplasia 2B syndrome due to codon 917 mutation: clinical manifestation and course in early and late onset disease. World J Surg.

[14] Bergholm U, Berstrom R, Ekbom A (1997). Long-term follow-up of patients with medullary carcinoma of the thyroid. Cancer.

[15] Brierley J, Tsang R, Simpson WJ (1996). Medullary thyroid cancer: analysis of survival and prognostic factors and the role of radiation therapy in local control. Thyroid.

[16] Dottorinin ME, Assi A, Sironi M (1996). Multivariate analysis of patients with medullary carcinoma: prognostic significance and impact on treatment of clinical and pathologic variables. Cancer.

[17] Clark JR, Friedman TR, Odell MJ (2005). Prognostic variables and calcitonin in medullary thyroid cancer. Laryngoscope.

[18] Gharib H, McConahey WM, Tiegs RD (1992). Medullary thyroid carcinoma: clinicopathologic features and long-term follow-up of 65 patients treated during 1946 through 1970. Mayo Clin Proc.

[19] Pilaete K, Delaere P, Decallonne B (2012). Medullary thyroid cancer: prognostic factors for survival and recurrence, recommendations for the extent of lymph node dissection and for surgical therapy in recurrent disease. B-ENT.

[20] Machens A, Dralle H (2013). Prognostic impact of N staging in 715 medullary thyroid cancer patients: proposal for a revised staging system. Ann Surg.

[21] Simoes-Pereira J, Bugalho MJ, Limbert E (2016). Retrospective analysis of 140 cases of medullary thyroid carcinoma followed-up in a single institution. Oncol Lett.

[22] Siironen P, Hagstrom J, Maenpaa HO (2016). Lymph node metastases and elevated postoperative calcitonin: predictors of poor survival in medullary thyroid carcinoma. Acta Oncol.

[23] Momin S, Chute D, Burkey B (2017). Prognostic variables affecting primary treatment outcome for medullary thyroid cancer. Endocr Pract.

[24] Randle RW, Balentine CJ, Leverson GE (2017). Trends in the presentation, treatment, and survival of patients with medullary thyroid cancer over the past 30 years. Surgery.

[25] Miyauchi A, Onishi T, Morimoto S (1984). Relation of doubling time of plasma calcitonin levels to prognosis and recurrence of medullary thyroid carcinoma. Ann Surg.

[26] Kameyama K, Takami H (2004). Medullary thyroid carcinoma: nationwide Japanese survey of 634 cases in 1996 and 271 cases in 2002. Endocr J.

[27] Ito Y, Miyauchi A, Yabuta T (2009). Alternative surgical strategies and favorable outcomes in patients with medullary thyroid carcinoma in Japan: experience of a single institution. World J Surg.

[28] Jung KY, Kim SM, Yoo WS (2016). Postoperative biochemical remission of serum calcitonin is the best predictive factor for recurrence-free survival of medullary thyroid cancer: a large-scale retrospective analysis over 30 years. Clin Endocrinol.

[29] Lee CR, Lee S, Son H (2016). Medullary thyroid carcinoma: a 30-year experience at one institution in Korea. Ann Surg Treat Res.

[30] Kwon H, Kim WG, Sung TY (2014). Changing trends in the clinicopathological features and clinical outcomes of medullary thyroid carcinoma. J Surg Oncol.

[31] Kim JH, Pyo JS, Cho WJ (2017). Clinicopathological significance and prognosis of medullary thyroid microcarcinoma: a meta-analysis. World J Surg.

[32] Miyauchi A, Matsuzuka F, Hirai K (2000). Unilateral surgery supported by germline RET oncogene mutation analysis in patients with sporadic medullary thyroid carcinoma. World J Surg.

[33] Miyauchi A, Matsuzuka F, Hirai K (2002). Prospective trial of unilateral surgery for nonhereditary medullary thyroid carcinoma in patients with germline RET mutations. World J Surg.

[34] Kihara M, Miyauchi A, Kudo T (2016). Reference values of serum calcitonin with calcium stimulation tests by electrochemiluminescence immunoassay before/after total thyroidectomy in Japanese patients with thyroid diseases other than medullary thyroid carcinoma. Endocr J.

[35] Kihara M, Miyauchi A, Kudo T (2017). Serum calcitonin reference values for calcium stimulation tests by electrochemiluminescence immunoassay in Japanese men with non-medullary thyroid carcinoma. Surg Today.

[36] Kudo T, Miyauchi A, Ito Y (2007). Diagnosis of medullary thyroid carcinoma by calcitonin measurement in fine-needle aspiration biopsy specimens. Thyroid.

[37] Wells SA, Gosnell JE, Gagel RF (2010). Vandetanib for the treatment of patients with locally advanced or metastatic hereditary medullary thyroid cancer. J Clin Oncol.

[38] Kurzrock R, Sherman SI, Ball DW (2011). Activity of XL184 (Cabozantinib), an oral tyrosine kinase inhibitor, in patients with medullary thyroid cancer. J Clin Oncol.

[39] Ito Y, Onoda N, Ito KI (2017). Sorafenib in Japanese patients with locally advanced or metastatic medullary thyroid carcinoma and anaplastic thyroid carcinoma. Thyroid.

[40] Ito Y, Tomoda T, Uruno T (2006). Prognostic significance of extrathyroid extension of papillary thyroid carcinoma: massive but not minimal extension affects the relapse-free survival. World J Surg.

[41] Kihara M, Miyauchi A, Yoshida H (2014). Tandem germline RET mutations in a family pathogenetic for multiple endocrine neoplasia 2B, confirmed by a natural experiment. Eur Thyroid J.

[42] Machens A, Hofmann C, Hauptmann S (2007). Locoregional recurrence and death from medullary thyroid carcinoma in a contemporaneous series: 5-year results. Eur J Endocrinol.

